# Requirements for innate immune pathways in environmentally induced autoimmunity

**DOI:** 10.1186/1741-7015-11-100

**Published:** 2013-04-04

**Authors:** Kenneth Michael Pollard, Dwight H Kono

**Affiliations:** 1 Department of Molecular and Experimental Medicine, The Scripps Research Institute, La Jolla, CA 92037, USA; 2 Department of Immunology and Microbial Science, The Scripps Research Institute, La Jolla, CA, 92037, USA

**Keywords:** Autoimmunity, Environment, Innate immunity, Lupus, mercury, Pristane, Silica, Type I interferon

## Abstract

There is substantial evidence that environmental triggers in combination with genetic and stochastic factors play an important role in spontaneous autoimmune disease. Although the specific environmental agents and how they promote autoimmunity remain largely unknown, in part because of diverse etiologies, environmentally induced autoimmune models can provide insights into potential mechanisms. Studies of idiopathic and environmentally induced systemic autoimmunity show that they are mediated by common adaptive immune response genes. By contrast, although the innate immune system is indispensable for autoimmunity, there are clear differences in the molecular and cellular innate components that mediate specific systemic autoimmune diseases, suggesting distinct autoimmune-promoting pathways. Some of these differences may be related to the bifurcation of toll-like receptor signaling that distinguishes interferon regulatory factor 7-mediated type I interferon production from nuclear factor-κB-driven proinflammatory cytokine expression. Accordingly, idiopathic and pristane-induced systemic autoimmunity require both type I interferon and proinflammatory cytokines whereas the less aggressive mercury-induced autoimmunity, although dependent on nucleic acid-binding toll-like receptors, does not require type I interferon but needs proinflammatory cytokines. Scavenger receptors and the inflammasome may contribute to silica-induced autoimmunity. Greater understanding of the innate mechanisms responsible for idiopathic and environmentally induced autoimmunity should yield new information into the processes that instigate and drive systemic autoimmunity.

## Review

Immunity requires contributions from both the innate and adaptive arms of the immune system. The innate component, found in all classes of plant and animal life, is hard-wired to recognize and respond rapidly to pathogens, but does not confer long-lasting or protective immunity [1]. In mammals, it is essential for the activation of the evolutionarily younger adaptive immune response [2], which, unlike the innate system, can be modified to generate highly specific antibodies and T cells capable of targeting virtually all foreign antigens. Adaptive immunity also mediates immunological memory, which facilitates faster, more effective responses to previously encountered antigens. Under normal circumstances, the immune system protects against infectious organisms, requiring it to distinguish foreign agents, including viruses, bacteria, fungi and parasites, from the host's healthy tissues. Failure to distinguish foreign from host, however, can result in the development of autoimmune diseases, including organ-specific disease with restricted tissue involvement, such as multiple sclerosis and type I diabetes, or more systemic involvement such as systemic lupus erythematosus (SLE). For most idiopathic autoimmune diseases, components of both the innate and adaptive immune responses are needed [3-5]. To varying extents, environmental factors also contribute to the development of autoimmunity. However, although idiopathic and environmentally induced systemic autoimmunity share common requirements [6,7], it is becoming clear that differences exist [8]. In this review we compare and contrast the innate immune system requirements for idiopathic systemic autoimmunity with systemic autoimmunity induced by exposure to mercury and pristane. We also discuss the innate immune components in silica-induced inflammatory responses that may contribute to silica-induced autoimmunity.

### Innate immunity

The innate immune response provides an immediate response to infection and injury and is mainly mediated by circulating factors and non-lymphocytic cell types that include macrophages, dendritic cells (DCs), neutrophils and other less common leukocytes. Surveillance mechanisms involve secreted, cell surface and intracellular pattern recognition receptors, such as toll-like receptors (TLRs), NOD-like receptors and RIG-I-like receptors [4,9]. Pattern recognition receptors respond not only to pathogen-associated molecular patterns, including bacterial and viral nucleic acids, lipoproteins and carbohydrates, but also to host-derived damage-associated molecular patterns such as ATP, high-mobility group box 1 and self-DNA. The recognition by pattern recognition receptors of these pathogen-associated molecular patterns and/or damage-associated molecular patterns results in cell signaling and activation of transcription factors such as NF-κB and IFN regulatory factors (IRFs) [10]. The resulting gene expression includes cytokines and chemokines, leading to inflammatory cell recruitment and activation, and expression of co-stimulatory molecules crucial for the induction of adaptive immunity [2].

### Innate immunity in idiopathic systemic autoimmunity

Systemic autoimmunity is thought to result from a mix of genetics, environmental factors and stochastic events [6]. Given the multitude of susceptibility genes, symptoms and immunological abnormalities, it is clear that numerous pathogenic pathways contribute to systemic autoimmune disease [5,11,12]. A major thrust of systemic autoimmunity research has centered on elucidation of abnormalities in the adaptive immune response [13,14]. However more recent research has identified the innate immune response as a major player in the initiation and expansion of systemic autoimmune pathology [4,5,9,15,16].

The current paradigm for the disease process of idiopathic systemic lupus-like autoimmunity argues for a central role of type I IFN [15,17,18]. This is based on the early observation of increased expression of IFN-α inducible genes (or IFN signature) in the peripheral blood cells of patients with SLE [17]. The type I IFN signature is found in 60% to 70% of patients with SLE, dermatomyositis, polymyositis or systemic sclerosis [19] but less frequently in patients with rheumatoid arthritis or multiple sclerosis [20]. The central role of type I IFN in SLE places special focus on the role of cells of the innate immune system, especially DCs [9,15,21]. DCs can be divided into three categories, conventional, plasmacytoid (pDC) and monocyte-derived [22]. DCs act as antigen presenting cells, are migratory and can control T cell responses [23]. Upon stimulation, pDCs produce large amounts of type I IFN in humans and mice and they are considered the main source of type I IFN in systemic autoimmunity [24]. Deletion of DCs, including pDCs, in lupus-prone MRL-*Fas*^*lpr*^ mice reduces disease severity including lymphocyte expansion, anti-chromatin autoantibodies and glomerulonephritis [25]. Most significantly, lupus-predisposed mice lacking pDCs due to the absence of IRF8 or showing pDC-specific defects in type I IFN production due to mutation of peptide/histidine transporter solute carrier family 15, member 4 do not develop autoimmunity [26]. These recent findings implicate pDCs and their ability to produce type I IFN as major contributors to the pathogenesis of lupus.

The important role that monocytes and macrophages play in phagocytosis, cytokine production and antigen presentation has also identified them as influential players in the innate immune response in systemic autoimmunity [27]. Deficiency of colony-stimulating factor-1, the principal growth factor for macrophages, in MRL-*Fas*^*lpr*^ mice reduces disease severity [28]. Deficiency of macrophage migration inhibitory factor reduces macrophage recruitment and glomerulonephritis in MRL-*Fas*^*lpr*^ mice [29]. Cultured in the presence of serum, macrophages from MRL-*Fas*^*lpr*^ mice have dysregulated gene expression compared with non-lupus-prone mice [30]. The presence of the complement component C1q also enhances immune-complex-mediated gene expression in monocytes of patients with SLE [31]. C1q preferentially promotes binding of immune complexes to monocytes rather than pDCs and thus indirectly reduces type I IFN production by pDCs [32]. The ability of C1q to suppress type I IFN may be an additional reason that C1q deficiency enhances susceptibility to SLE [33].

Type I IFN plays a significant role in the onset and severity of idiopathic autoimmunity. Induction of type I IFN by TLR3 and melanoma differentiation-associated protein-5 agonist, polyinosinic:polycytidylic acid (poly(I:C)), exacerbates idiopathic systemic autoimmunity, particularly nephritis, in C57BL/6-*Fas*^*lpr*^[34], NZW hybrid [35] and NZB/NZWF1 [36] mice. Moreover sustained production of type I IFN by injection of adenovirus-expressing IFN-α also exacerbates disease, including glomerulonephritis in idiopathic lupus models [37-40]. By contrast, deficiency of type I IFN receptor (IFNAR) reduces disease severity in most autoimmune models [41,42] except MRL-*Fas*^*lpr*^, where absence of IFNAR leads to more severe disease [43] and antibody blocking of the IFNAR has minimal beneficial effect [44]. The mechanism whereby IFNAR deficiency exacerbates disease in MRL-*Fas*^*lpr*^ mice is unknown, however, deletion of DCs (including pDCs) in this model while still allowing T and B cell activation, hypergammaglobulinemia and anti-nucleolar autoantibodies (ANoA) does substantially reduce disease severity [25], suggesting that DCs are required for promoting autoimmune disease by mechanisms beyond the production of type I IFN.

Type I IFN expression relies on activation of TLRs and signaling via IRF7 [4,45]. Numerous studies have determined that endosomal TLRs, particularly TLR7 and TLR9, influence idiopathic systemic autoimmunity [46-49]. However, specific endosomal TLRs make different contributions to disease severity. Loss of TLR3 does not impact disease [50] although TLR3 stimulation of myeloid differentiation factor 88 (MyD88)-deficient mice, which lack TLR7 and TLR9 signaling, partially recovers disease [51]. Absence of TLR7 partially ameliorates disease [52-54] whereas deficiency of TLR9 exacerbates autoimmunity in a TLR7-dependent manner [52,53]. The 'triple D' mutation in Unc-93 homolog B1 (*Unc93b1)*, an integral component of endoplasmic reticulum, involved in the trafficking of TLR3, TLR7 and TLR9 [55,56], abolishes endosomal TLR signaling [55] and suppresses disease in lupus-prone C57BL/6-*Fas*^*lpr*^, BXSB [46] and MRL-*Fas*^*lpr*^ (Koh YT et al, J. Immunol. In press).

All TLR signaling pathways lead to activation of the transcription factor NF-κB and production of proinflammatory cytokines (for example, IL-1, IL-6, TNFα) [4,57,58]. Accordingly, modulation of certain individual proinflammatory cytokines can have significant effects on the expression of idiopathic autoimmunity. For example, treatment with anti-IL-6 or anti-IL-6 receptor antibody results in reduced severity of kidney damage in lupus-prone mice [59,60] whereas recombinant IL-6 exacerbates glomerulonephritis [61]. Complete deficiency of IL-6 in MRL-*Fas*^*lpr*^ mice reduces clinical, immunological and histological indices of lupus and improves survival [62]. IL-1, which consists of α and β forms [63], is elevated in idiopathic lupus models [64,65]. Treatment with recombinant IL-1 receptor [66] reduces the severity of systemic autoimmunity as does IL-1 receptor antagonist [67], although the latter appears not to be effective against established disease [68]. The contributions of the separate α and β forms of IL-1 remain to be determined. The role of another proinflammatory cytokine, TNFα, in systemic autoimmunity is less clear. Treatment with TNFα increases survival in lupus-prone mice [69,70] and loss of *Tnf*[71] or *Tnf* receptors [72] accelerates disease. By contrast, treatment with anti-TNF receptor increases survival [73].

hese studies show that innate immune responses contribute significantly to disease severity in idiopathic systemic autoimmunity. Principal contributions, identified to date, come from pDCs and TLR- or IRF7-mediated type I IFN production. However it is clear that proinflammatory cytokines, especially IL-6, expressed by TLR or NF-κB signaling also play a significant role. In addition early components of the complement cascade are protective.

### Innate immunity in environmentally induced systemic autoimmunity

That systemic autoimmunity can be elicited by exogenous factors, especially medications, is well established in both humans and animal models [6,74,75]. These can trigger disease in individuals with or without susceptibility to idiopathic autoimmunity or can lead to enhancement of existing autoimmune disease. However, these observations come with two clear caveats. First, there are no accepted criteria for diagnosis or classification of environmentally associated autoimmunity in humans, nor are there criteria that distinguish environmentally associated autoimmunity from types of idiopathic autoimmune diseases [76]. Second, although studies of animal models have provided critical understanding of many facets of human systemic autoimmunity [12], they are limited by incomplete representation of the full spectrum of human disease [77]. Nonetheless, common mechanisms of adaptive immunity exist for both induced and idiopathic disease in human and animals including loss of tolerance, T and B cell activation and autoantibody production [6,78]. However, the role that innate immunity plays is only beginning to be examined. Exposure to environmental agents such as mercury [79-81], crystalline silica [82,83] and pristane [84] are known to result in a lupus-like systemic autoimmunity in animal models. Although the mechanisms of induction are poorly understood, published as well as our preliminary studies suggest that specific environmental triggers induce or modulate systemic autoimmunity through distinct components of the innate immune system.

### Pristane

Pristane, also known as 2,6,10,14-tetramethylpentadecane (or TMPD), is a component of mineral oil that induces chronic inflammation and plasmacytomas in mice [85]. In humans, mineral oil or petroleum waste has been associated with rheumatoid arthritis and possibly lupus [84]. In susceptible strains of mice, pristane injection causes a lupus-like disease characterized by a wide spectrum of primarily antinuclear autoantibodies (ANA) and immune complex-mediated glomerulonephritis [84]. Severity of disease including IgG autoantibodies and glomerulonephritis are reduced in the absence of IFN-γ [86], IL-6 [87] and IL-12p35 [88]. Pristane-induced autoimmunity may also fall under a common syndrome called ASIA (autoimmune syndrome induced by adjuvants) [89].

Similar to SLE, pristane-induced autoimmunity is associated with increased expression of type I IFN-inducible genes in peripheral blood cells (IFN signature) [90,91]. The most severe aspects of disease are dependent on type I IFN; type I IFN receptor-deficient (*Ifnar*^−/−^) mice exposed to pristane exhibit markedly reduced lupus-specific autoantibodies, proteinuria and glomerular hypercellularity [92]. Type I IFN expression, autoantibody production and glomerulonephritis in pristane-treated mice are primarily mediated via a TLR7- and MyD88-dependent pathway [93,94]. In addition, deficiencies in TLR4 and TLR9 also impact disease severity [95]. Interestingly, TLR deficiency differentially affects lupus-specific autoantibody production, with absence of TLR7 or TLR9 reducing anti-ribonucleoprotein responses but not anti-DNA [94,95] whereas lack of TLR4 reduced production of both anti-ribonucleoprotein and anti-DNA autoantibodies [95]. Pristane-treated *Ifnar*^−/−^ mice also have reduced expression and activation of TLR7 and TLR9 in B cells [96], suggesting a positive feedback mechanism in which type I IFN augments TLR-mediated B cell responses. In contrast to spontaneous lupus, type I IFN production in pristane-induced autoimmunity is not dependent on DCs, but is produced by immature Ly6C^high^ inflammatory monocytes [97]; increases in Ly6C^high^ monocyte numbers correlates with greater amounts of lupus-specific autoantibodies [97]. Type I IFN is also required for the chemokine expression necessary for recruitment of inflammatory monocytes [98], which likely results in a positive feedback signal and further acceleration of IFN production. This expansion of monocytes by type I IFN appears to be relatively specific because the lack of the inflammatory cytokines TNF-α, IL-6, IL-1 [98] and IFN-γ, which are required for disease [84], have no effect on Ly6C^high^ monocyte recruitment.

The chronic inflammatory response to pristane also includes neutrophil infiltration, which in contrast to monocytes requires IL-1, specifically IL-1α, and is mediated by MyD88 and IL-1 receptor-associated kinase, but not IRF7 [99]. IL-1β, caspase 1 and the inflammasome components NOD-like receptor family, pyrin domain containing 3 (NLRP3) and apoptosis-associated speck-like protein containing a CARD (ASC) (which are required for caspase 1 activation [100]) are not required for neutrophil recruitment in pristane-induced chronic inflammation [99]. Although IL-1alpha; has not been directly linked to the autoimmunity elicited by pristane, it does induce expression of IL-6 [101], which is required for pristane-induced hypergammaglobulinemia and the production of anti-DNA and anti-chromatin [87].

The protein product of IRF5, a lupus susceptibility gene [102], acts as a transcription factor to mediate TLR induction of proinflammatory cytokines IL-6, IL-12, TNFα and, to some extent, IFN-α, independent of NF-κB [103-105]. Notably, *Irf5* deficiency reduced pristane-induced disease severity including the expansion of Ly6C^high^ monocytes, type I IFN signature, autoantibodies and renal disease [106-109]. This was confirmed in pure *Irf5*-deficient mice lacking a spontaneous *Dock2* mutation found in some Irf5 knockout lines that alters pDC and B cell development and type I IFN production [108-110].

These studies suggest that disease expression and severity in the pristane model are tightly linked to nucleic acid-sensing TLR and MyD88 signaling leading to type I IFN production analogous to idiopathic lupus. Unlike idiopathic lupus, however, type I IFN production is produced by immature monocytes rather than pDCs. By contrast, although a feature of this model is chronic inflammation, inflammasome components and IL-1β appear to play little if any role.

### Mercury

Exposure to mercury in humans has been associated with autoimmune manifestations in small surveys, but more definitive large-scale epidemiological studies are lacking [111]. Studies of South American gold miners documented that mercury exposure was associated with higher levels of proinflammatory cytokines (IFN-γ, TNF-α, IL-1β) and autoantibodies [80,112]. In other studies, mercury exposure from skincare products was associated with membranous nephropathy [79,113]. Thus, although only limited human populations at potential risk for mercury-induced autoimmunity have been studied in detail [75,113], the severity of systemic disease that was induced by mercury exposure appears mild compared to that of idiopathic SLE.

Possible mechanisms for mercury-induced systemic autoimmunity have come largely from studies of susceptible mice and rats that, when exposed to mercury, develop lymphocyte activation, ANA and deposits of immune complexes in blood vessels and glomeruli [77]. The adaptive immune responses required for murine mercury-induced autoimmunity (mHgIA) share common requirements with idiopathic lupus including certain cytokines [114,115], co-stimulation factors [116,117] and transcription factors [8,118]. However, whereas type I IFN signaling pathways predominate in idiopathic and pristane-induced autoimmune disease, our recent studies indicate that mHgIA is independent of type I IFN. Accordingly, *Ifnar1*-deficient C57BL/6, NZB and BXSB mice all have similar autoimmune responses to mercury exposure as wild-type mice (Kono and Pollard, unpublished observations), in contrast to the known type I IFN-dependence of spontaneous autoimmunity in both NZB and BXSB strains [41,44]. This lack of dependence on type I IFN is further supported by the observation that mercury-induced hypergammaglobulinemia and autoantibodies in *Inept* mice, which are deficient in IRF7 and consequently do not produce IFN-α after TLR7 or TLR9 stimulation [119], are not reduced compared to the wild-type (Kono and Pollard, unpublished observations).

Similar to idiopathic [47,53] and pristane-induced autoimmunity [93-95], severity of mHgIA is impacted by TLR activation because the TLR4 ligand lipopolysaccharide exacerbates disease [120]. Although the specific TLRs required for mHgIA remain to be examined, mercury-exposed autoimmune-prone BXSB mice with the triple D mutation in *Unc93b1* (required for endosomal TLR3, TLR7 and TLR9 signaling [121]) do not develop ANA or increased serum IgG unlike wild-type BXSB (Kono and Pollard, unpublished observations). Thus, although endosomal TLRs contribute to mHgIA, type I IFN is not required in both autoimmune-prone and healthy genetic backgrounds. It is possible that mercury exposure can replace type I IFN by activating the IFNAR pathway or related genes downstream of IFNAR activation. Alternatively, mHgIA may not be mediated by type I IFN. Related to this, our preliminary studies show that mercury exposure suppresses IFN-α induction mediated by poly(I:C) (TLR3 agonist) while proinflammatory cytokine (for example, IL-6) production is unaffected (Kono and Pollard, unpublished observations). This supports the latter possibility that mHgIA is not mediated by type I IFN and may also explain why mHgIA is a relatively mild disease compared to idiopathic lupus [19,80]. It remains to be determined, however, if mHgIA can be exacerbated by exogenous type I IFN. The lack of dependence on type I IFN but requirement for endosomal TLRs is similar to spontaneous lupus in the MRL background [43,53].

Endosomal TLR signaling leads to cell activation and to type I IFN production via IRF7 and the induction of proinflammatory cytokines IL-6, pro-IL-1β and TNF-α via the NF-κB pathway [4,122-124]. Dependence on endosomal TLRs but not *Irf7* or *Ifnar* suggests that mHgIA may be primarily mediated by NF-κB signaling [4]. IL-1 signaling also activates NF-κB [101] and we have shown that cell bound IL-1α is required for mercury-induced T cell proliferation *in vitro*[125], suggesting that cell signaling via the IL-1 receptor may also be important for mHgIA. We have, however, shown that neither NLRP3 nor caspase 1 deficiency impacts expression of mHgIA [8], suggesting that IL-1β is not required. In other experiments, we also examined the effects of IL-6, which is induced by NF-κB [101], on mHgIA and found B10.S-*Il6*^−/−^ mice exposed to HgCl_2_ had reduced serum IgG autoantibodies and kidney deposits of IgG compared to wild-type mice [126]. Although the pathways have yet to be defined, taken together, these studies point to the endosomal TLRs, the proinflammatory cytokines IL-1α and IL-6 but not type I IFN as the major innate factors that drive autoimmunity following exposure to mercury. Furthermore, NF-κB-associated pathways, but not IRF7 were implicated.

### Silica

Silica exposure is common in mining, sandblasting, rock drilling, granite cutting, construction work, bricklaying and cement work. In 2007, the US Occupational Safety and Health Administration estimated that almost two million individuals in the USA are occupationally exposed to respirable crystalline silica [127] and exposure continues to be a national and worldwide problem [128]. Inhalation of crystalline silica can cause silicosis, which is characterized by chronic inflammation and scarring in the upper lobes of the lungs [128]. Additionally, epidemiologic data have repeatedly associated silica exposure with systemic autoimmunity [111] including SLE, rheumatoid arthritis and systemic sclerosis [83,111,129-131]. Notably, silica dust exposure is associated with high titers of ANA [132] and both the presence of autoantibodies and clinical symptoms are positively correlated with intensity (that is, concentration and frequency) of exposure [133,134]. Further support has come from animal models in which lupus in susceptible mice is exacerbated by exposure to silica [135,136] and ANAs develop in non-autoimmune mice and rats exposed to silica products [137,138]. The mechanisms mediating silica-induced autoimmunity are not yet defined. Nevertheless, one possibility is that the chronic inflammatory milieu present in silicosis might induce or exacerbate autoimmunity through the production of proinflammatory cytokines and release of self-antigens [139-141].

Silica-induced inflammation is mainly caused by the toxic effects of silica on alveolar macrophages, resulting in the release of proinflammatory chemokines and cytokines including TNF and IL-1 [128,142], and the influx of neutrophils, macrophages, DCs and lymphocytes [143-145]. Silica-induced pulmonary inflammation is dependent on IFN-γ [146] but not Th2 cytokines such as IL-4 and IL-13 [147], or IL-12 [148], requirements similar to those of mHgIA [8,114]. Innate immunity mediates this process as silica-induced inflammation and fibrosis can occur in the absence of T, B, NKT or NK cells [143]. Notably, although acute lung inflammation requires IL-17 [149], chronic inflammation is dependent on type 1 IFN and IRF7 [150]. NALP3 (NACHT, LRR and PYD domains-containing protein 3) inflammasome components, caspase-1 and IL-1β, are also required for silicosis [142,151-153] and our preliminary findings indicate that caspase-1 is required for autoantibody induction (Kono and Pollard, unpublished observations). Although the role of individual TLRs has not been examined, silica has been shown to suppress TLR-mediated activation of DCs [144], but its effect on TLR stimulation of alveolar macrophages, the primary cell-type responsible for inflammasome-mediated lung inflammation [142], is not known. Death of alveolar macrophages by silica might further promote inflammation and autoimmunity by impairing the clearance of silica and apoptotic cells, and by generating apoptotic material. In support of this, deficiency of either scavenger receptors macrophage receptor with a collagenous structure (MARCO) or CD204, expressed mainly on macrophages, was shown to impair silica clearance and exacerbate silica-induced lung inflammation [154,155]. Additionally, MARCO-deficient mice are defective in clearing apoptotic cells [156] and both MARCO and CD204 have been argued to promote tolerance to apoptotic cell material [157]. These observations suggest that scavenger receptor-mediated uptake of silica and subsequent macrophage cell death may adversely affect clearance of dead and dying cells, which, in turn, could impact self-tolerance [158,159] and promote autoimmunity.

Studies with silica-induced lung inflammation, while only indirectly implying mechanisms of silica-induced autoimmunity, suggest that, like idiopathic lupus and pristane-induced autoimmunity, innate mechanisms involving IRF7 and type I IFN might play pivotal roles. Silica-induced killing of scavenger receptor-bearing macrophages, inflammasome activation and IL-1β are also likely to make significant contributions. The requirement for the inflammasome pathway is clearly different from the innate immune responses required for the development of pristane-induced autoimmunity [84,99] and mHgIA [8].

### Innate immune mechanisms contributing to environmentally induced autoimmunity

As is clear from the studies discussed above, innate immunity plays an essential role in both idiopathic and environmentally induced lupus-like autoimmunity, with the requirement for endosomal TLRs and/or Unc93b1 providing a unifying mechanism for idiopathic and pristane- and mercury-induced disease [4]. Signaling by these TLRs leads to cell activation and the production of proinflammatory cytokines via NF-κB and type I IFNs by IRF7 activation [4]. By contrast, as presented above, different innate pathways have been implicated in the development of pristane-, mercury- and silica-induced autoimmune diseases that mediate the induction of inflammation, cell death, the adaptive response and autoimmunity, supporting our contention that environmental factors can induce or enhance lupus-like autoimmunity through several different innate mechanisms. How the different innate responses are elicited and how they consequently promote autoimmunity remains to be determined. However, a few possible explanations can be postulated.

Recent studies suggest that adaptor protein complex 3 (AP-3), which is involved in the sorting of transmembrane proteins to lysosomes and lysosome-related organelles (LRO), may bifurcate these signaling pathways because AP-3 is required for TLR7 and TLR9 induction of type I IFN but not proinflammatory cytokines [56]. AP-3 mediates trafficking of TLRs and UNC93B1 to the lysosome-associated membrane protein 2+ (LAMP2+) late endosomes and LROs but not to vesicle-associated membrane protein 3+ (VAMP3+) early endosomes [4,56]. Thus UNC93B1-mediated endosomal TLR trafficking moves to early endosomes in an AP-3 independent manner, leading to NF-κB-regulated proinflammatory cytokine production (NF-κB endosome), and then in an AP-3 dependent step to late endosomes/LRO and IRF7-mediated type I IFN production (IRF7 endosome) [4]. The importance of endosomal location in DC responses has been shown by studies using different classes of CpG oligonucleotide ligands to stimulate TLR9 signaling [160-162]. In addition, viperin, a component of endoplasmic reticulum-derived lipid storage granules or lipid bodies, is required for endosomal TLR-mediated type I IFN by pDCs but does not contribute to proinflammatory cytokine production in pDCs or type I IFN production by other cell types [163]. Viperin may thus be central to the role of pDCs and type I IFN production in systemic autoimmunity.

The bifurcation of TLR trafficking and signaling regulated by AP-3 may explain the dependence of mHgIA on Unc93b1 and proinflammatory cytokines like IL-6 as well as its type I IFN independence. Mercury is known to accumulate in lysosomes [164,165] and may thus impact AP-3 controlled TLR trafficking to the IRF7 endosome, leading to reduced type I IFN production. Silica can also affect lysosomal function, particularly in macrophages [166,167], suggesting that silica-induced autoimmunity may also be influenced by effects on lysosome function. We can find no evidence that pristane affects lysosome function, however given the type I IFN-dependence of pristane-induced autoimmunity [92], we expect pristane not to hamper TLR trafficking and/or signaling.

**Figure 1:**
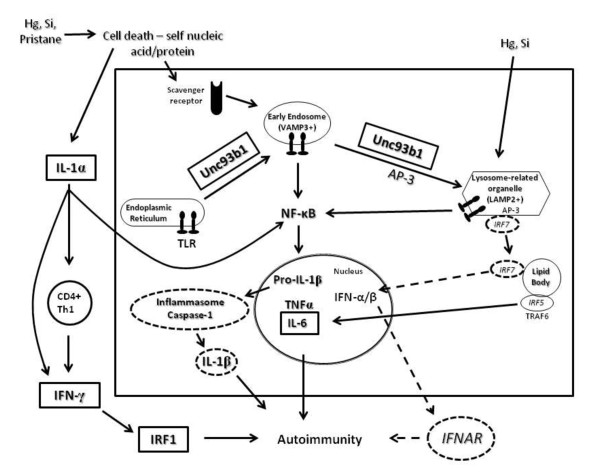
**Innate immune mechanisms contributing to environmentally induced autoimmunity.** The toxic response to environmental agents results in self nucleic acid/protein complexes that may become ligands for endosomal TLRs via scavenger receptors, particularly in macrophages. UNC93B1-mediated trafficking of endosomal TLRs leads first to VAMP3+ early endosomes, where signaling results in NF-κB activation and proinflammatory cytokine production. TLRs, again in concert with Unc93b1, also traffic to LAMP2+ LROs where IRF7 is activated to stimulate type I IFN expression. Lipid bodies, which contain components of the TLR signaling complex, may contribute to type I IFN particularly in pDCs. Activation of IRF5 in complex with TRAF6 can lead to proinflammatory cytokine production. NF-κB-mediated proinflammatory cytokine production may be augmented by release of constitutively expressed IL-1α from dead and dying cells. IL-1α may also contribute to adaptive immunity via differentiation and expansion of CD4+ T cells and enhanced expression of IFN-γ-stimulated genes such as *IRF1*. The large box signifies signaling events in innate immune responses that may occur in one or more cell types. Steps required for mHgIA are shown in rectangles with a thick black line while those not required are shown by ovals with a broken line. Steps required for pristane-induced autoimmunity include those leading to type I IFN and proinflammatory cytokine production and may also include pathways involving IL-1α, particularly IL-1α-driven NF-κB activation. AP-3, adaptor protein complex 3; Hg, mercury; IFN, interferon; IFNAR, type I IFN receptor; IL, interleukin; IRF, interferon regulatory factors; LAMP2, lysosome-associated membrane protein 2; LRO, lysosome-related organelle; NF, nuclear factor; Si, silica; Th1, T helper type 1; TLR, Toll-like receptor; TNF, tumor necrosis factor; TRAF6, TNF receptor associated factor 6; UNC93B1, Unc-93 homolog B1; VAMP3, vesicle-associated membrane protein 3.

In Figure 1 we outline our view of the mechanisms of innate immunity in environmentally induced autoimmunity with emphasis on the contribution of bifurcation of TLR signaling to mHgIA. We propose that the toxic response to mercury [168], pristane [169] or silica [170] leads to the availability of nucleic acid/protein self-antigens. These are then brought into the endolysosomal machinery of antigen presenting cells such as DCs, macrophages and/or B cells where they complex with TLRs and traffic to early endosomes (NF-κB endosome), leading to NF-κB-regulated proinflammatory cytokine production. IRF7-mediated type I IFN production via late endosomes/LRO (IRF7 endosome) has little role in mHgIA as suggested by the failure of *Ifnar* and *Irf7* deficiency to suppress mHgIA. The important role of the mercury-induced NF-κB-mediated inflammatory response is likely aided by IL-1α from dead and dying cells. IL-1α is also important for mercury-induced T cell proliferation [125] and may contribute to enhanced CD4+ T cell expansion and differentiation [171]. In addition, IL-1α synergizes with IFN-γ to regulate IFN-γ induced gene expression in an NF-κB-dependent manner [172], thus linking the innate and adaptive responses in mHgIA. Interestingly, IL-1α, released from dying cells, can initiate sterile inflammation involving neutrophils [173], supporting its role in granulocyte recruitment in pristane-induced chronic inflammation [99]. We would argue that, unlike pristane, mHgIA may not require NF-κB independent proinflammatory cytokine production mediated by IRF5, particularly as IRF5 requires TNF receptor-associated factor 6 (TRAF6) [103], which is a component of the signaling complex of lipid bodies [4].

## Conclusions

Innate immunity plays an essential role in both idiopathic and environmentally induced autoimmunity, however there are clear differences in the required molecular and cellular components that mediate disease development. In idiopathic autoimmunity, both type I IFN and proinflammatory cytokines are needed for disease with pDCs being the primary cells involved in type I IFN production. By contrast, in pristane-induced autoimmunity, TLR/MyD88 signaling, leading to type I IFN and proinflammatory cytokines, does not require DCs, but rather immature monocytes. Mercury-induced autoimmunity, although showing clear evidence of TLR involvement does not require type I IFN, but rather shows significant dependence on proinflammatory cytokines such as IL-1α and IL-6. Additional pathways may apply to silica-induced autoimmunity as scavenger receptors and the inflammasome are central to silica-induced inflammatory responses. It can be speculated that some of these differences may be related to the bifurcation of TLR signaling that distinguishes IRF7-mediated type I IFN production and NF-κB-driven proinflammatory cytokine expression. These findings from several environmentally induced models suggest that environmental triggers can induce autoimmunity through diverse innate pathways. A greater understanding of the specific innate processes that initiate or exacerbate disease will be key to understanding the role of environmental factors in autoimmunity.

## Abbreviations

AP-3: Adaptor protein complex 3; DC: Dendritic cells; IFN: Interferon; IFNAR: Type I IFN receptor; Hg: Mercury; Ig: Immunoglobulin; IL: Interleukin; IRF: Interferon regulatory factors; LAMP2: Lysosome-associated membrane protein 2; LRO: Lysosome-related organelles; MARCO: Macrophage receptor with a collagenous structure; mHgIA: Murine mercury-induced autoimmunity; MyD88: Myeloid differentiation factor 88; NF: Nuclear factor; NK: Natural killer; pDC: Plasmacytoid dendritic cell; Si: Silica; SLE: Systemic lupus erythematosus; TLR: Toll-like receptor; TNF: Tumor necrosis factor; TRAF6: TNF receptor associated factor 6; Unc93b1: Unc-93 homolog B1; VAMP3: Vesicle-associated membrane protein 3.

## Competing interests

The authors declare that they have no competing interests.

## Authors' contributions

This review is an expanded version of a presentation given at the 8th International Congress on Autoimmunity in Granada, Spain in 2012. KMP and DHK designed the review and concepts and drafted the manuscript. Both authors read and approved the final manuscript.

## Funding

This work was funded by the National Institutes of Health grants ES014847 and ES020388 to KMP and AR053731 and AR060181 to DHK.

## Pre-publication history

The pre-publication history for this paper can be accessed here:

http://www.biomedcentral.com/1741-7015/11/100/prepub
